# Comparative Evaluation of role of Lysyl oxidase gene (LOXG473A) expression in pathogenesis and malignant transformation of Oral Submucous Fibrosis

**DOI:** 10.4317/jced.55980

**Published:** 2019-10-01

**Authors:** Akhilanand Chaurasia, Neetu Singh, Dinesh Sahu, Archna Mishra

**Affiliations:** 1 Assistant professor. Department of Oral Medicine & Radiology. Faculty of Dental Sciences. King George’s Medical Unniversity Lucknow; 2 Associate Professor. Molecular Biology Unit, Center for Advance Research. King George’s Medical University, Lucknow; 3 Post doctoral Fellow. Molecular Biology Unit. Center for Advance Research. King George’s Medical University, Lucknow; 4 PhD Scholar. Molecular Biology Unit, Center for Advance Research. King George’s Medical University, Lucknow

## Abstract

**Background:**

Identification and comparison of gene expression of Lysyl oxidase (LOX) in oral submucous fibrosis and controls and to determine its role in Pathogenesis of Oral submucous fibrosis.

**Material and Methods:**

Of total sample size (n=127), the whole blood sample were collected from case and control group in citrate vial. It is centrifused and stored at -800C. We collected and isolated RNA from blood of case group (n=127) and age and sex matched control group (n=127) recruited on the basis of inclusion criteria. The cDNA was prepared for 127 samples which were processed for gene expression of Lysyl oxidase (LOX) in relation to housekeeping genes (Beta actin and 18srRNA) and its role in pathogenesis of Oral submucous fibrosis.

**Results:**

In relative expression (Normalized ratio),relatively 11 cases shown down-regulation of lysyl oxidase gene while 27 cases shows up-regulation of lysyl oxidase gene while in 89 cases there were no regulation i.e expression of lysyl oxidase gene in case group was of same degree of control. In non-relative expression results (Non-normalized ratio), the 38 cases shown down regulation of LOX gene while in 53 cases, it was up-regulated however in remaining 36 cases there was neither up-regulation nor down-regulation of Lysyl oxidase gene i.e the expression of LOX gene is null.

**Conclusions:**

In oral submucous fibrosis, the expression of Lysyl oxidase gene is mixed type i.e either it will down regulate/upregulate or there will be no expression at all comparatively. However in majority of cases the upregulation of lysyl oxidase is relatively more common than down-regulation or non expression of Lysyl oxidase gene.

** Key words:**Oral submucous fibrosis, lysyl oxidase, betel nut, premalignant disorders.

## Introduction

Oral Submucous fibrosis is a chronic debilitating disease and a potentially malignant condition of oral cavity. The incidence of oral squamous cell carcinoma has been estimated to be 7.6% in oral submucous fibrosis patients after 17 years follow up (1). There are 200-400 million areca nut chewers worldwide. Areca nut associated oral squamous cell carcinoma is the 3rd most common malignancy in developing countries (2). Oral submucous fibrosis is associated exclusively with areca nut chewing and is believed to be a homeostatic disorder of extracellular matrix (3). Epidemiological evidences strongly indicate association of the betel quid habit and oral submucous fibrosis. The areca nut (betel nut) component of betel quid especially an alkaloid called arecoline plays a major role in pathogenesis of oral submucous fibrosis by causing an abnormal increase in the collagen production. The synthesis of collagen is influenced by a variety of mediators, prominent mediator being transforming growth factor-beta (TGF-beta). The TGF-beta1 in particular seems to be the one that plays a major role in wound repair and fibrosis. It causes the deposition of extra-cellular matrix by increasing the synthesis of matrix proteins like collagen and decreasing its degradation by stimulating various inhibitory mechanisms (1). TGF- beta has been found to strongly promote the expression of Lysyl oxidase (LOX) both at mRNA and protein levels in various cell lines (1). The LOX is dependent on copper for its functional activity (1).Arecanuts have been shown to have a high copper content and chewing areca nuts for 5 to 30 minutes significantly increases soluble copper levels in oral fluids. The increased level of soluble copper could act as an important factor in oral submucous fibrosis by stimulating fibrogenesis through upregulation of LOX activity (4). Previous studies have specified the upregulation of LOX expression in normal oral keratinocytes by arecanut extract and its oncogenesis for oral squamous cell carcinoma (5). There are 7 single nucleotide polymorphism (SNP) sites in LOX coding region including C225G,G409C,G473A,C476A,G816A,T924G and A1135G2. Among all the SNP sites in LOX coding region G473A has the highest polymorphic frequency. The genetic susceptibility or diseases nature of oral submucous fibrosis is still largely undefined2.The present study is designed to investigate the presence of LOXG473A gene expression in Oral submucous fibrosis patients and its role in development of oral cancer.

## Material and Methods

The study subjects (n=127) were recruited from the OPD Department of Oral medicine and Radiology, King George Medical University, Lucknow, India. A structured questionnaire was used to record all necessary data. The ethical clearance is obtained from Institutional Ethics Committee of King George Medical University, Lucknow, India. The study subjects having clinically and histopathologically diagnosed oral submucous fibrosis, betel quid chewing habit without clinical evidence of oral submucous fibrosis and healthy individuals without betel quid chewing habit and having normal oral mucosa were included in study however the patients who are on regular analgesics or anti-inflammatory drugs which are known to interfere with Lysyl oxidase activity and patients who have already received or on treatment for oral submucous fibrosis is excluded from study. The study population is divided in 2 groups. Group 1 includes study subjects with clinically and histopathologically proven oral submucous fibrosis (Case group) and group 2 includes study subjects without Oral submucous fibrosis (Control group). A written consent was obtained from each participant in study. The whole blood sample was collected from case and control group in citrate vial. It was centrifused and stored at -800C. We collected and isolated RNA from blood of case group (n=127) and age and sex matched control group (n=127) recruited on the basis of inclusion criteria.

a. RNA extraction. This study was done by isolating RNA by using trizol method which is mentioned below-

Take 200 ul of blood and 1 ul trizol in 1.5 ml tube and vortex it for 2 min → Keep it on room temperature for 2 min→ Now spin the sample on 12000g for 10 minutes at 4 degree→ Collect the supernatant→ Add 250 ul chloroform→ Mix it 200 times by hand→ Keep on RT for 15 min→ Spin at 12000g for 10 min→3 layers will appear (upper Aqueous layer=RNA, Middle white layer=DNA, Bottom layer=Protein) → Collect upper Aqueous layer→ Add 500 microlitre of isopropanol in other tube→ Mix 100 times by hand→ Spin at 12000g for 15 mins→ Discard the supernatant(Take another tube and transfer collection in it ) → Add 500 microlitre of 70% alcohol→ Spin at 12000g for 10 mins→ Add 500 microlitre of 70% alcohol→ Spin at 12000g for 10 mins→ Discard the supernatant and spin for 1 min→ Remove extra liquid by pipette→ Keep it for 5 min at RT→ Add 25microlitre of RNAse free water→ Keep it for 20 min at RT→ Store it at -20 degree.

The cDNA was prepared for 127 samples which were processed for gene expression of Lysyl oxidase (LOX) in relation to housekeeping genes (Beta actin and 18srRNA) and its role in pathogenesis of Oral submucous fibrosis.

b. cDNA Synthesis. The first strand cDNA will be synthesized by annealing 2 μg of RNA with 0.5 μg of oligo d(T)18 primer in a total reaction volume of 20 μl using M-MLV reverse transcriptase. The mixture will be incubated for one hour at 37 °C, further the enzyme will be deactivated by heating at 95 °C for five minutes. Targeted and housekeeping gene specific primers will be validated through gradient PCR and will be used for real-time PCR amplifications (ABI-Step One).

c. Real Time PCR. Briefly, 0.5 μl cDNA, 10 pmol of each PCR-validated primers and 10 μl SYBR Green master mix in a final volume of 20 μl with a negative control (no template) will be subjected to activation step at 95 °C for 10 min, followed by 40 cycles of : 15 s at 95 °C, 15 s at the Tm (specific for the primer pairs used), 1min at 59°C with a single fluorescence measurement. After the amplification phase, a melting curve cycle will be set at 95 °C for 15 s, 59 °C for 1 min with a continuous measurement to confirm later about the amplification of a single product. Real time PCR will be repeated thrice for each sample. The relative level of each transcript in different sample will be calculated by normalization of the value with the corresponding reference and will also be compared with the Ct values of positive calibrator.

## Results

Of total sample size (n=150), the whole blood sample were collected from case and control group in citrate vial. It is centrifused and stored at -800C. We collected and isolated RNA from blood of case group (n=127) and age and sex matched control group (n=127) recruited on the basis of inclusion criteria. The cDNA was prepared for 127 samples which were processed for gene expression of Lysyl oxidase (LOX) in relation to housekeeping genes (Beta actin and 18srRNA) and its role in pathogenesis of Oral submucous fibrosis. The crossing point, Ct values were acquired for both the target and reference gene using ABI Stepone RT-PCR software. The relative level of each transcript in different samples was calculated by normalization of the value with the corresponding reference and compared among them using Ct values for Case 1 cDNA as positive calibrator. Comparison of the relative expression level of each transcript was analyzed by REST 2009 software with 2000 time iterations.

**Figure 1:**
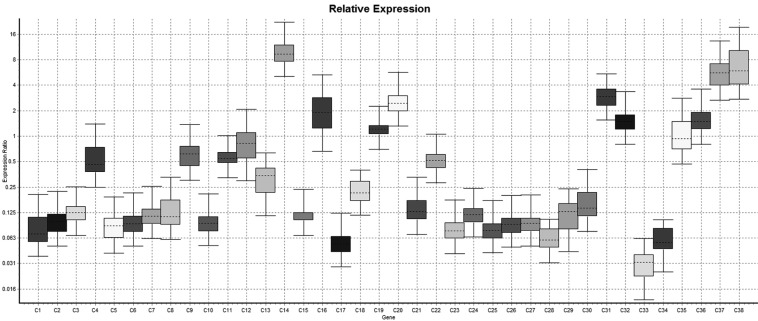
Whisker plot diagram shows comparative relative expression of the LOX gene in samples no 1 to 38.

**Figure 2:**
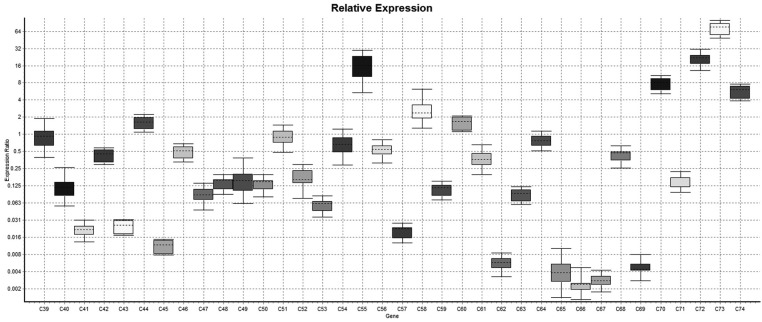
Whisker plot diagram shows comparative relative expression of the LOX gene in samples no 39 to 74.

**Figure 3:**
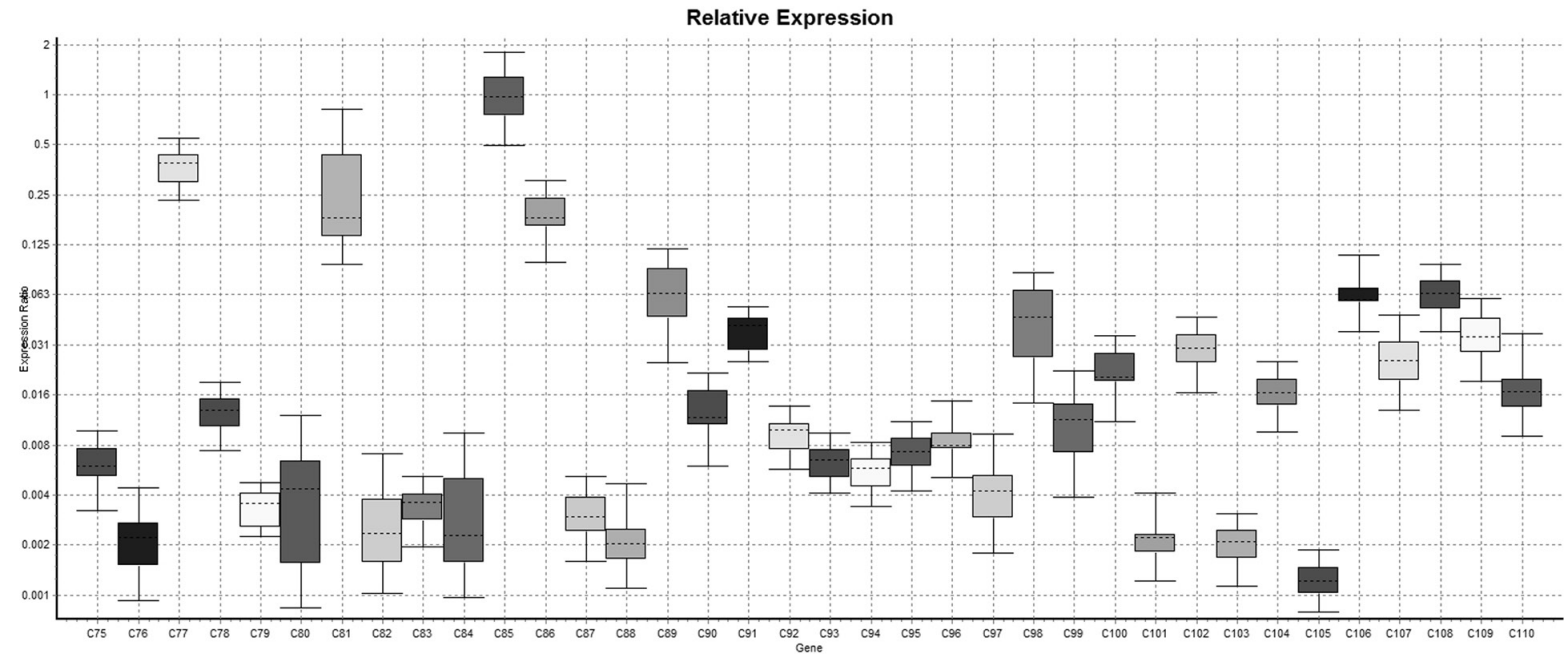
Whisker plot diagram shows comparative relative expression of the LOX gene in samples no 75 to 110.

**Figure 4:**
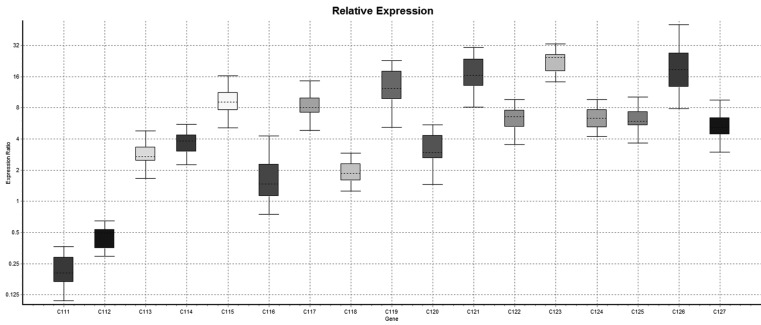
Whisker plot diagram shows comparative relative expression of the LOX gene in samples no 111 to 127.

In relative expression (Normalized ratio),relatively 11 cases shown down-regulation of lysyl oxidase gene while 27 cases shows up-regulation of lysyl oxidase gene while in 89 cases there were no regulation i.e expression of lysyl oxidase gene in case group was of same degree of control. So it was concluded that in case group the up-regulation of lysyl oxidase was more common than down-regulation comparatively. However either of upregulation or down regulation is statistically significant (*p*<.001). In non-relative expression results (Non-normalized ratio), the 38 cases shown down regulation of LOX gene while in 53 cases, it was up-regulated however in remaining 36 cases there was neither up-regulation nor down-regulation of Lysyl oxidase gene i.e the expression of LOX gene is null. The down-regulation and up-regulation of lysyl oxidase gene was statistically significant (*P*<.001) (Figs. 1-4).

In oral submucous fibrosis, the expression of Lysyl oxidase gene is mixed type i.e either it will down regulate/up-regulate or there will be no expression at all comparatively. However in majority of cases the upregulation of lysyl oxidase is relatively more common than down-regulation or non expression of Lysyl oxidase gene. So the expression of lysyl oxidase gene can be used as adjunctive diagnostic tool for oral submucous fibrosis.As the expression of lysyl oxidase gene is mixed type in our study, we are not in agreement with previous studies that the lysyl oxidase gene is only upregulated. We cannot definitely say its role in pathogenesis of in oral submucous fibrosis however more studies are needed in this field to reach a definite conclusion.

## Discussion

Oral submucous ﬁbrosis (OSF) is characterized by a general ﬁbrotic change in the oral submucosa and the progressive tissue rigidity resulting in diffculties in mouth-opening, tongue movement, intolerance to spicy food and rigidity of the lip (7). OSF is associated exclusively with areca chewing and was believed to be a homeostatic disorder of extracellular matrix (ECM) (8). OSF is predominately seen in people in South Asian countries, or South Asian (9). It is now a public health issue in many parts of the world, including the United Kingdom (10), South Africa (11) and many South East Asian countries (12,13).

The common hypothesis for OSF pathogenesis is the increase of collagen synthesis or reduced collagen degradation in oral sub-epithelial tissues because of chemical, physical or inﬂammatory irritation. Although the available epidemiological evidence indicates that the chewing of the areca nut is an important risk for developing of OSF (14-16). Not all chewers develop the disease. Other information, however, indicates that the disease is not necessarily dose-response related and genetic predisposition might explain such individual variability (16). Also, cessation of the habit also does not inﬂuence the characteristics of the disease once it is established (17). Diverse disruptions in inﬂammatory cytokines and ECM organizers are involved in the pathogenic pro- cesses. OSF patients expressed higher tissue inhibitors of metalloproteinases-1 (TIMP1) and lower matrix metal- loproteinases-2 (MMP2) cause stabilization of extra- cellular matrix (7,18,19). Tannins puriﬁed from areca can make collagen resistance to collagenase (20). The inﬂammatory effect of OSF is associated with COX-2 (21) which can induce prostaglandin as inﬂammatory mediator. Cytokines such as transforming growth factor (TGF)-b, IL-1, IL-6, platelet-derived growth factor (PDGF) and basic ﬁbroblast growth factor (bFGF) are up regulated in OSF than normal group (7,22). Arecoline, the major alkaloid in areca, can induce the synthesis of collagen (23). A recent report demonstrated that the treatment with collagenase reduced the symp- toms of OSF (24). In addition, genetic polymorphisms in TNF-a, MMP-3, major histocompatibility complex class I chain related gene A (MICA), and cytotoxic T- lymphocyte associated antigen 4 (CTLA-4), which are associated with ECM organization or inﬂammation are risk factors to OSF (25-28). Lysyl oxidase (LOX) is a copper- activated enzyme critical for collagen cross-linking and organization of extracellular matrix. The presence of a G to A polymorphism at nucleotide 473 caused a non- conservative Arg158Gln change in the LOX amino acid sequence. OSF is a precancerous lesions characterized by the accumulation of collagen in oral submocousa (29). Shieh *et al.* (30), conducted a study on Taiwan population to find the association between LOX gene polymorphism and OSMF in older male areca chewers. The presence of G to A polymorphism at nucleotide at 473, caused a non-conservative Arg158Gln change in the LOX amino acid sequence. They investigated the relationship between LOX Arg158Gln polymorphism and risk of OSMF. There was a borderline statistical significant difference in Arg158Gln genotype between control and OSMF patients. However, the G/A + A/A of LOX Arg158Gln in OSMF patients older than 50 years was statistically significantly higher than controls below 50 years. In study conducted by Shieh *et al.*, (30) on Taiwan population, the mean duration of areca chewing habit in patients less than 50 years and more than 50 years was 15 years and 21 years, respectively. Shieh *et al.*, (31) identified an upregulation of LOX and LOXL2 mRNA expression in areca-associated OSCC tissues and cell lines relative to their normal counterparts. Other previous investigations that focused mostly on breast carcinomas, identified that LOX can induce migration and invasion of malignant breast epithelial cells. Shieh *et al.* (30) in his study included sample size of 83 OSMF patients and 216 areca chewers without OSMF as control. In the present study, the sample size included was 20 OSMF patients and 20 areca chewers without OSMF. The absence of LOX gene polymorphism in the present study could also be attributed to the smaller sample size. This necessitates further studies with larger sample size.Another possible explanation for the variation in the LOX gene polymorphism between Taiwan population and Indian population could be the difference in genetic makeup.

LOX is a cuproenzyme that is essential for stabilization of extracellular matrixes, specifically the enzymatic cross-linking of collagen and elastin. Copper is essential for organic cofactor formation in lysyl oxidase (32).

Areca products contain high levels of copper (mean 302 nmol/g). Hence local factors such as composition of the quid, consistency of quid, duration and frequency of habit and whether saliva is expelled or swallowed after chewing, can affect the uptake of copper into the oral epithelium (33). These factors may explain the marked variations seen in the copper levels within the OSMF group which in turn may affect the regulation of LOX gene and its polymorphism in OSMF patients. However, in the present study these factors were not considered.LOX coding region contains seven single nucleotide polymorphism (SNP) sites that includes C225G, G409C, G473A, C476A, G816A, T924G and A1135G. Among all the SNP sites in LOX coding region, G473A has the highest polymorphic frequency (30).

These results demonstrate that LO expression is upregulated in OSF as in other fibrotic diseases such as fibrosis of the liver. In the OSF cases the distribution of LO positivity was closely associated with collagen in the juxta-epithelial lamina propria and submucosa and was identified as a secreted protein localized mostly in the extracellular matrix. It is therefore feasible to suggest that the excessive deposition of heavily cross-linked collagen seen in OSF may be impart due to an increase in the activity of LO. Collagen deposition in the early stages of fibrogenesis in murine schistosomiasis is associated with upregulation of LO (34).

The mechanism of this upregulation by fibroblasts in OSF is not fully understood. Cytokine mediators such as IL-1, TGF-b and EGF have been shown to increase LO activity in smooth muscle cultures (35) and immunohistochemically detectable levels of IL-1a and b , IL-6, IFN, TGF-b,PDGF and FGFb have been shown to be increased in OSF tissues (36).

Our data show an inverse relationship between the severity of the fibrosis and the expression of LO. In early cases of OSF, as measured by mouth opening more than 30 mm, there is intense LO staining and this tends to decrease as the severity of fibrosis progresses. This may suggest that LO expression is related to active elastin and collagen syn- thesis early in the disease process. In cases with mouth opening below 30 mm, the level of LO immunopositivity is lower suggesting that much of the fibrosis has already taken place. This would support the hypothesis that the accumulation of collagen seen in ad- vanced cases of OSF is not only due to fibrogenesis, but may also be due to a decrease in the breakdown of the colla- gen earlier cross-linked by lysyl oxidase, and thus a reduced overall turnover. It would be desirable to look at LO expression in earlier stages of OSF, such as in blanching of mucosa, to confirm these findings (33).

It has been shown that the amount of dietary or cellular copper can markedly influence the functional activity of LO (37,38) and that levels of dietary copper intake influence skin lysyl oxidase in young men39. It is therefore possible that the excess copper from areca nut may upregulate LO activity in oral mucosa, resulting in fibrosis. The precise mechanism by which this occurs is not fully understood. LO has a relatively short half-life and it is expressed transiently, as shown in developing granulomas in murine schistosomiasis (34).

One possible explanation is that the enzyme activity may be stabilised by copper, thus increasing its half-life. Sec- ondly, at the molecular level, the N-terminus of exon 1 of the LO molecule has copper binding sites, and this interaction may upregulate the expression of the enzyme at the cellular level. Further studies are in progress to investigate LO activity of oral fibroblasts following ex posure to copper in vitro.Based on our previous observations that copper is released from areca prod- ucts during chewing and is deposited in oral tissues, taken together with our current findings that LO activity is up- regulated in OSF, we hypothesize that these cellular events lead to cross- linking of collagen and elastin, thus making them less degradable. The upregulation of LO in OSF may be an important factor in the pathogenesis and progression of this disorder and merits further detailed study (33).
